# Microstructure Evolution and Strength–Toughness Trade-Off in 1Cr12Ni2WMoVNb Martensitic Stainless Steel During Tempering

**DOI:** 10.3390/ma19153350

**Published:** 2026-08-06

**Authors:** Jiashun Gao, Liehua Liu, Zhilong Xu, Jiang Zhu, Zhijie Hu, Hongxin Lin

**Affiliations:** 1 School of Marine Mechatronics, Xiamen Ocean Vocational College, Xiamen 361000, China; gaoshunjia@163.com (J.G.);; 2 Engineering Research Center of Anti-Fatigue Manufacturing for Marine Equipment of Fujian Province, Xiamen 361000, China; 3 School of Marine Equipment and Mechanical Engineering, Jimei University, Xiamen 361000, China; 4 Chengyi College, Jimei University, Xiamen 361000, China; 5 Sichuan Huadian Gongxian Power Generation Co., Ltd., Yibin 644500, China

**Keywords:** tempering temperature, 1Cr12Ni2WMoVNb, martensitic stainless steel, mechanical properties, secondary hardening

## Abstract

The influence of tempering temperature on the microstructure evolution and mechanical properties of 1Cr12Ni2WMoVNb martensitic stainless steel was systematically investigated in the range of 200–650 °C. Microhardness, tensile strength, and impact toughness were evaluated, combined with analyses using confocal laser scanning microscopy (CLSM), scanning electron microscopy (SEM), transmission electron microscopy (TEM), energy-dispersive spectroscopy (EDS), and phase characterization techniques. The results show that hardness and tensile strength initially decrease, then increase to a peak at approximately 550 °C due to secondary hardening, and finally decline significantly. In contrast, impact toughness exhibits an inverse trend, revealing a clear strength–toughness trade-off. Microstructural analysis indicates that this behavior is governed by the competition among multiple strengthening mechanisms. At intermediate temperatures, the precipitation of fine and dispersed M23C6 carbides leads to strong dislocation pinning and pronounced precipitation strengthening, resulting in peak strength but reduced plastic deformation capability. At higher temperatures, carbide coarsening and dislocation recovery weaken strengthening while restoring plasticity, thereby improving toughness. These findings clarify the temperature-dependent competition among strengthening mechanisms governing the strength–toughness evolution during tempering.

## 1. Introduction

1Cr12Ni2WMoVNb martensitic stainless steel exhibits an excellent combination of strength, hardness, toughness, and corrosion resistance due to its Cr–Ni-based alloying system and alloying additions of Mo, V, and Nb. These alloying elements not only improve the comprehensive mechanical properties but also strongly influence carbide precipitation behavior and microstructural evolution during tempering. Compared with conventional martensitic stainless steels, such as 13Cr4Ni and CA6NM steels, 1Cr12Ni2WMoVNb possesses a more complex alloying design, resulting in complex interactions among precipitation strengthening, solid-solution strengthening, and dislocation evolution during tempering. Owing to its outstanding comprehensive properties, this steel has been widely used in critical components such as rifle barrels, pressure vessels, valves, steam turbine blades, gas turbines, and aero-engine compressor blades [1,2,3]. Therefore, understanding its temperature-dependent microstructural evolution and mechanical response is important for optimizing heat treatment and achieving an appropriate balance between strength and toughness.

For 1Cr12Ni2WMoVNb martensitic stainless steel, the typical quenching process involves heating the material to 950–1050 °C and holding at this temperature to ensure complete and homogeneous austenitization, followed by rapid oil cooling to obtain a martensitic microstructure [4]. During rapid cooling, the martensitic transformation is accompanied by volume expansion and lattice distortion, which generate considerable residual tensile stresses within the material [5]. These stresses may promote crack initiation after quenching and reduce structural stability. Therefore, tempering treatment is essential to relieve quenching-induced residual tensile stresses and optimize both microstructure and mechanical properties. According to the temperature range, tempering is generally classified into low-temperature tempering, medium-temperature tempering, and high-temperature tempering [6]. Low-temperature tempering slightly reduces strength but provides only limited improvement in toughness, whereas high-temperature tempering significantly decreases strength while remarkably enhancing toughness, thereby improving the overall service reliability of the material [7].

Extensive and systematic studies have been conducted on the tempering behavior of martensitic stainless steels. Previous studies have shown that with increasing tempering temperature, tensile strength generally decreases, while impact toughness gradually improves [8]. Further analysis indicates that high-temperature tempering promotes carbide precipitation, which reduces the carbon content in lattice interstitial sites and consequently weakens the solid-solution strengthening effect [9]. In addition, both the martensitic structure and precipitated carbides tend to coarsen at elevated temperatures, further influencing the mechanical properties [10,11,12]. Dislocation evolution is also significantly affected: as the tempering temperature increases, dislocation density gradually decreases, making crystal slip easier and reducing the material’s resistance to plastic deformation [13]. Other studies have reported that under sensitization tempering conditions, hardness first increases and then decreases with prolonged tempering time [14]. Particularly in the tempering range of 500–600 °C, a large number of carbides, mainly M_3_C and M_2_C, precipitate in the steel. Among them, the finely dispersed M_2_C carbides can produce a pronounced secondary hardening effect, enabling the material to achieve its maximum hardness [15].

As an advanced martensitic stainless steel, it is necessary to systematically investigate the influence of tempering temperature on the evolution of mechanical properties and the transition of strengthening mechanisms in 1Cr12Ni2WMoVNb steel. In this study, hardness, tensile strength, and impact toughness were evaluated under different tempering temperatures. Combined with microstructural characterization, energy-dispersive spectroscopy (EDS), transmission electron microscopy (TEM), fracture morphology and phase analysis, the influence of tempering temperature on strength evolution was systematically investigated, and the contributions and competition of different strengthening mechanisms were discussed. This work provides a theoretical basis for optimizing the heat treatment process of this material for high-end equipment manufacturing applications.

## 2. Materials and Methods

### 2.1. Materials

**Figure 1:**
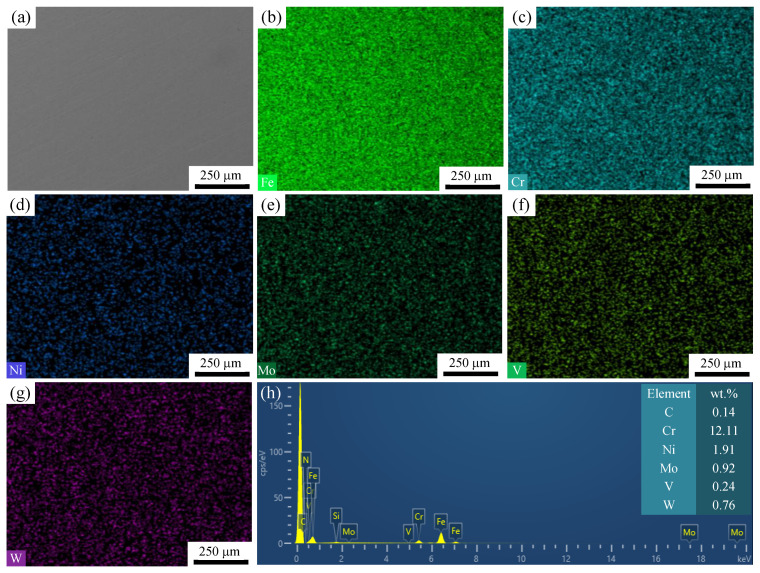
EDS analysis of the material: (**a**) selected analysis area; (**b**) Fe element distribution; (**c**) Cr element distribution; (**d**) Ni element distribution; (**e**) Mo element distribution; (**f**) V element distribution; (**g**) N element distribution; (**h**) elemental contents.

The experimental material used in this study was 1Cr12Ni2WMoVNb martensitic stainless steel, and its chemical composition is listed in Table 1. The EDS results indicate that the distribution of alloying elements in the material was relatively uniform, as shown in Figure 1a–g. The measured chemical composition satisfied the required specifications, as presented in Figure 1.

The specimen preparation procedure was as follows. First, billets with dimensions of 20 × 40 × 150 mm were placed in a vacuum quenching furnace (model HZQ-80, Huaxiang, Beijing, China). The samples were heated to 1050 °C at a rate of 20 °C/min and held at this temperature for 60 min to ensure complete and homogeneous austenitization. Subsequently, the specimens were immediately quenched in oil (model YJ002, Yingji, Suzhou, China) to obtain the martensitic microstructure. The same oil quenching condition was applied to all specimens before the subsequent tempering treatment.

### 2.2. Methods

**Figure 2:**
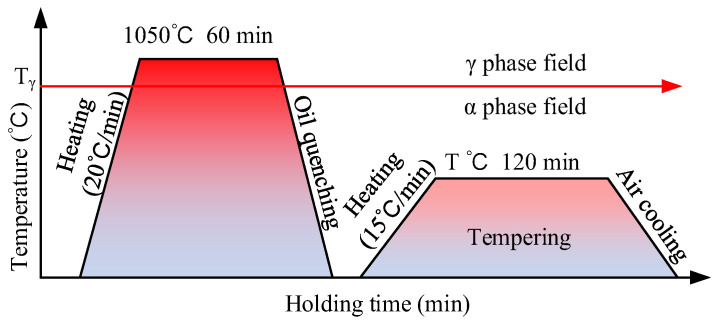
Schematic illustration of the quenching and tempering process of 1Cr12Ni2WMoVNb steel (T = 200 °C, 250 °C, 300 °C, 350 °C, 400 °C, 450 °C, 500 °C, 550 °C, 600 °C, and 650 °C).

The quenched specimens were cut by wire electrical discharge machining into ten groups for tempering treatment. A box-type tempering furnace (model HZR-20, Huaxiang, Beijing, China) was used to perform tempering treatment on the specimens at different temperatures. The selected tempering temperatures were 200 °C, 250 °C, 300 °C, 350 °C, 400 °C, 450 °C, 500 °C, 550 °C, 600 °C, and 650 °C, and the holding time was fixed at 2 h. After the holding process, all specimens were naturally cooled in air at room temperature (25 °C). The detailed heat treatment procedure is illustrated in Figure 2.

**Figure 3:**
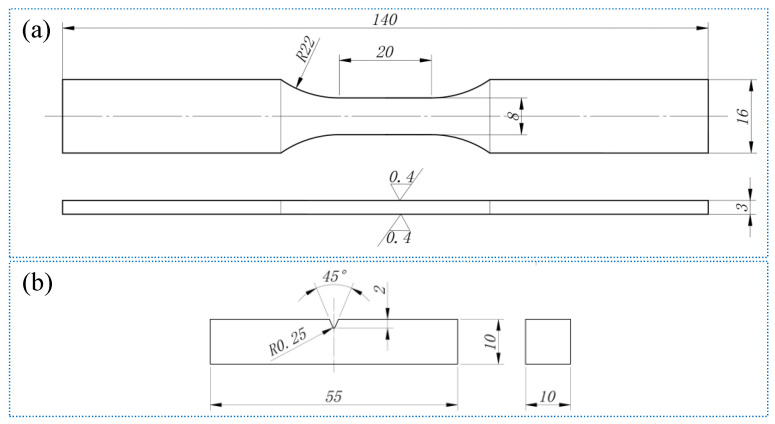
Schematic illustrations of mechanical testing specimens: (**a**) plate-shaped tensile specimen with a gauge length of 20 mm; (**b**) V-notched Charpy impact specimen.

After quenching and tempering, a series of characterizations and analyses were conducted, including mechanical property tests (Vickers microhardness, tensile properties, and impact toughness), microstructural characterization using CLSM, SEM, EDS, TEM, and XRD techniques, and fracture morphology characterization of impact specimens by SEM. The geometries and dimensions of the tensile specimens and Charpy impact specimens used in this study are shown in Figure 3.

To ensure the reliability and reproducibility of the experimental results, three independent specimens were prepared and tested for tensile properties and impact toughness under each tempering condition. For Vickers hardness measurements, three specimens were evaluated for each condition, and three randomly selected indentation points were measured on each specimen. The average value was calculated, and the standard deviation was used to characterize the data dispersion. Vickers hardness tests were performed using a microhardness tester (FALCON 500, Innovatest, Maastricht, The Netherlands) with a load of 100 gf (HV0.1) and a dwell time of 10 s. Tensile strength was evaluated using a universal tensile testing machine (model WDW-200, Precision Instrument Co., Shanghai, China) under constant displacement mode, with a crosshead speed of 0.5 mm/min. Impact toughness was tested using a Charpy impact testing machine (model JB-300B, Liling, Jinan, China).

Microstructural characterization was performed using a confocal laser scanning microscope (CLSM, model VK-X3000, Keyence, Osaka, Japan) and a field emission scanning electron microscope (FE-SEM, model Crossbeam 550, Zeiss, Oberkochen, Germany). EDS analysis was carried out using an Xplore 30 detector (Oxford Instruments, Abingdon, UK) attached to the Crossbeam 550 FE-SEM to identify elemental composition and distribution characteristics. TEM analysis was conducted using a transmission electron microscope (model JEM-2100F, JEOL, Tokyo, Japan). Phase analysis was performed using an X-ray diffractometer (model SmartLab 9, Rigaku, Tokyo, Japan).

The preparation procedure for metallographic specimens was as follows. First, cross-sectional samples were obtained by wire cutting. The samples were then mechanically ground sequentially using 400#, 800#, 1200#, 1500#, and 2000# SiC abrasive papers, followed by polishing with W1.5 diamond polishing paste. Finally, the specimen surfaces were etched using a stainless steel-specific etchant composed of 60 vol.% deionized water, 30 vol.% hydrochloric acid, and 10 vol.% ferric chloride for 10 s, followed by cleaning with absolute ethanol and air drying.

The preparation procedure for TEM specimens was as follows. First, samples were sectioned by wire cutting. They were then mechanically polished using abrasive papers and ultrasonically cleaned. Finally, thin foils for TEM observation were prepared using the focused ion beam (FIB) function of the Crossbeam 550 field emission SEM (ZEISS, Oberkochen, Germany) and subsequently mounted on copper grids.

## 3. Results

### 3.1. Mechanical Properties

**Figure 4:**
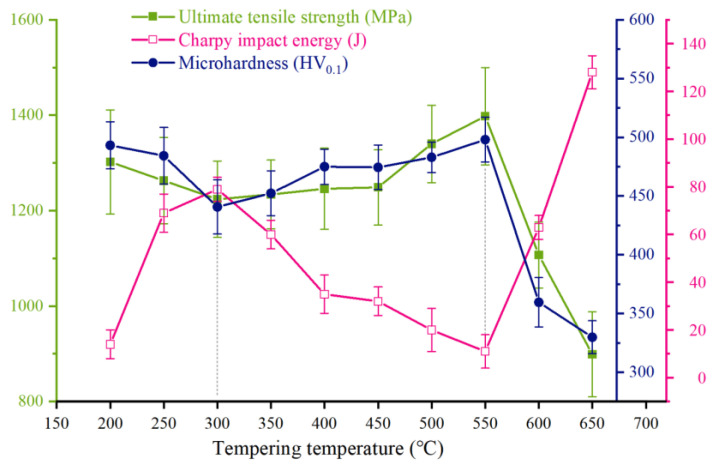
Variation in mechanical properties with increasing tempering temperature.

The experimental results indicate that both the microhardness and tensile strength of the material exhibited a similar evolution trend with increasing tempering temperature: they initially decreased slightly, then gradually increased to a secondary hardening peak, and finally declined rapidly. In contrast, the variation trend of impact energy was opposite, as shown in Figure 4.

At a tempering temperature of 550 °C, a pronounced secondary hardening phenomenon was observed, with the hardness reaching 498.01 HV_0.1_ and the tensile strength increasing to 1398.34 MPa. However, the impact energy was relatively low, measuring only 11.21 J. In addition, this temperature falls within the sensitization temperature range (≥400 °C), which may significantly reduce the corrosion resistance of the material. By comparison, under the tempering condition of 300 °C, the material maintained relatively high hardness (440.74 HV_0.1_) and tensile strength (1224.39 MPa), while exhibiting excellent impact toughness of 79.22 J, demonstrating a favorable balance between strength and toughness. Furthermore, this temperature effectively avoids the sensitization temperature range of martensitic stainless steel, which is beneficial for preserving the corrosion resistance of the material.

### 3.2. Microstructural Analysis

**Figure 5:**
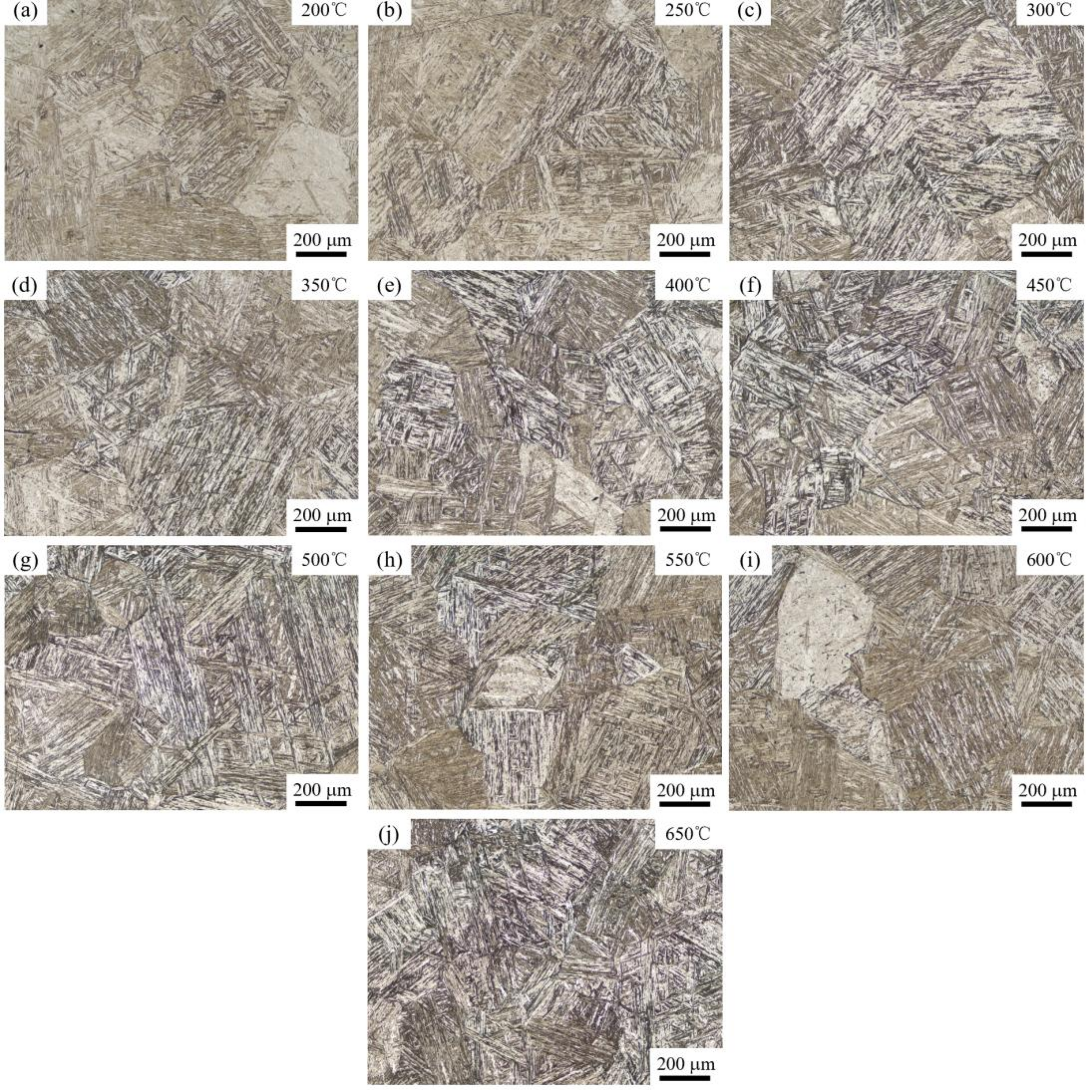
Microstructures observed by CLSM: (**a**) tempered at 200 °C; (**b**) tempered at 250 °C; (**c**) tempered at 300 °C; (**d**) tempered at 350 °C; (**e**) tempered at 400 °C; (**f**) tempered at 450 °C; (**g**) tempered at 500 °C; (**h**) tempered at 550 °C; (**i**) tempered at 600 °C; (**j**) tempered at 650 °C.

The metallographic specimens were examined using CLSM, and the corresponding results are presented in Figure 5. With increasing tempering temperature, the microstructure of the material underwent significant evolution. After tempering at 200 °C, 250 °C, and 300 °C, the microstructure gradually transformed into tempered martensite, as shown in Figure 5a–c. When the tempering temperature increased to 350 °C, 400 °C, 450 °C, and 500 °C, the microstructure further evolved into tempered troostite, as shown in Figure 5d–g. After tempering at 550 °C, 600 °C, and 650 °C, the microstructure was finally transformed into tempered sorbite, as shown in Figure 5h–j.

**Figure 6:**
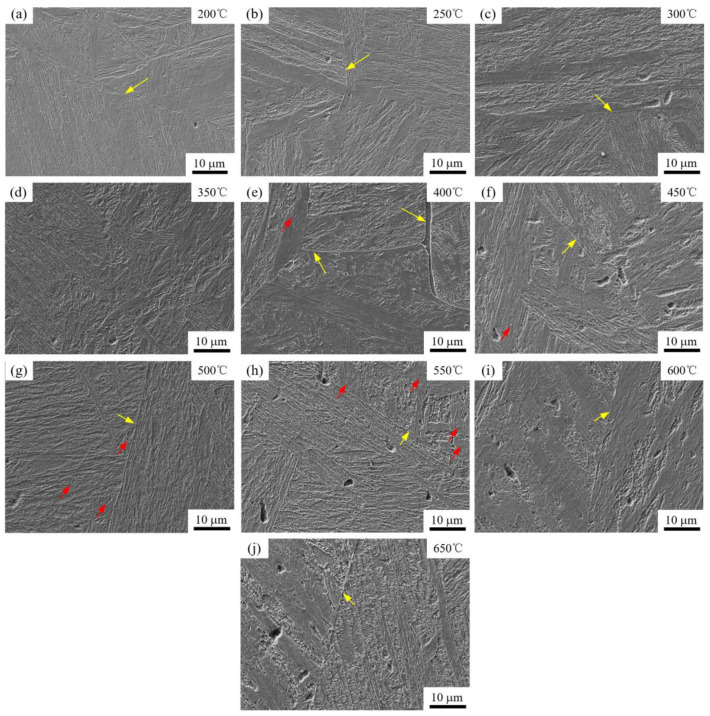
SEM microstructures: (**a**) tempered at 200 °C; (**b**) tempered at 250 °C; (**c**) tempered at 300 °C; (**d**) tempered at 350 °C; (**e**) tempered at 400 °C; (**f**) tempered at 450 °C; (**g**) tempered at 500 °C; (**h**) tempered at 550 °C; (**i**) tempered at 600 °C; (**j**) tempered at 650 °C. Yellow arrows indicate prior austenite grain boundaries, while red arrows indicate precipitated fine particles.

The microstructures were further characterized by SEM, and the results are shown in Figure 6. During the low-temperature tempering stage (200–300 °C), the microstructure mainly retained the lath martensitic morphology, exhibiting clear boundaries and sharp features, as shown in Figure 6a–c. As the tempering temperature increased to 350–550 °C, significant microstructural changes were observed. The boundaries of lath martensite gradually became indistinct, while carbide precipitation became increasingly pronounced. In particular, in the specimen tempered at 550 °C, fine and dispersed precipitate particles were observed in local regions, which correspond well to the secondary hardening phenomenon revealed by the tensile strength results. This indicates that this temperature range represents the dominant stage of precipitation strengthening by carbides, as shown in Figure 6d–h. In the high-temperature tempering range (600–650 °C), further coarsening of the microstructure and carbide agglomeration were observed. The martensitic characteristics nearly disappeared, and the microstructure gradually transformed into a relatively uniform tempered sorbite morphology, as shown in Figure 6i,j.

**Figure 7:**
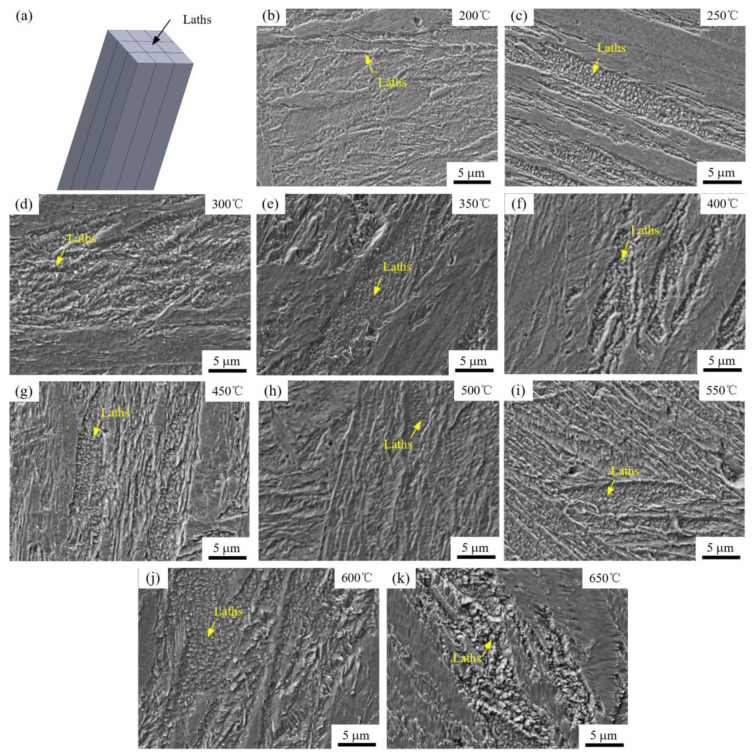
Analysis of lath structures in the microstructure: (**a**) schematic illustration of the martensitic lath structure; (**b**–**k**) lath-like microstructures after tempering at 200 °C, 250 °C, 300 °C, 350 °C, 400 °C, 450 °C, 500 °C, 550 °C, 600 °C, and 650 °C, respectively.

The metallographic specimens were examined by SEM. The lath regions were further magnified, and representative lath cross-sectional areas were selected, as shown in Figure 7. A schematic illustration of the selected region is provided in Figure 7a. Overall, with increasing tempering temperature, the martensitic laths gradually coarsened, and the initially well-defined lath boundaries became increasingly indistinct, as shown in Figure 7b–k. For the specimen tempered at 250 °C, the average lath cross-sectional size is approximately 300 nm, as shown in Figure 7c. In contrast, for the specimen tempered at 650 °C, the average lath cross-sectional size increases to approximately 800 nm, as shown in Figure 7k.

Overall, with increasing tempering temperature, the microstructure gradually evolved from lath martensite to tempered sorbite. Correspondingly, the strengthening mechanism shifted from dislocation strengthening and solid-solution strengthening to precipitation strengthening, and finally to microstructural softening. This evolution trend is highly consistent with the variation in tensile strength, revealing the intrinsic relationship between microstructural characteristics and mechanical properties during different tempering stages.

### 3.3. EDS Analysis

**Figure 8:**
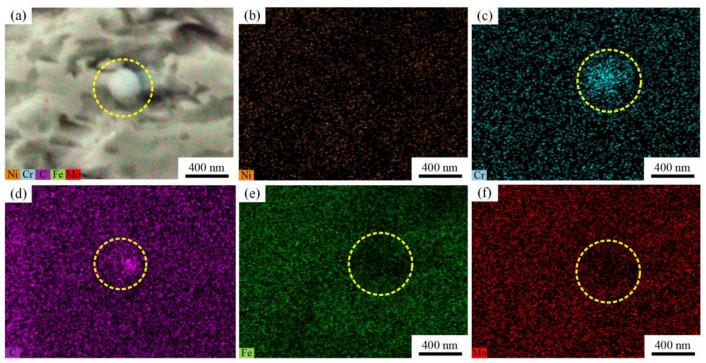
EDS analysis of the specimen tempered at 500 °C: (**a**) microstructure; (**b**) Ni element distribution; (**c**) Cr element distribution; (**d**) C element distribution; (**e**) Fe element distribution; (**f**) Mo element distribution. The yellow circles indicate the regions containing precipitated particles.

**Figure 9:**
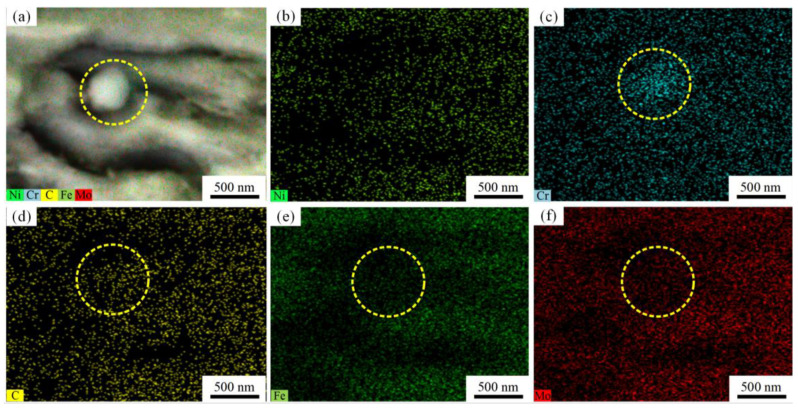
EDS analysis of the specimen tempered at 550 °C: (**a**) microstructure; (**b**) Ni element distribution; (**c**) Cr element distribution; (**d**) C element distribution; (**e**) Fe element distribution; (**f**) Mo element distribution. The yellow circles indicate the regions containing precipitated particles.

The microstructures of the specimens tempered at 500 °C and 550 °C were further examined at higher magnification using SEM, and a large number of fine precipitated particles with diameters ranging from 200 to 400 nm were observed, as shown in Figure 8 and Figure 9. In terms of overall particle size, the precipitates in the specimen tempered at 550 °C were noticeably larger than those in the specimen tempered at 500 °C, as shown in Figure 8 and Figure 9a.

EDS analysis revealed that these precipitated particles were significantly enriched in Cr, as shown in Figure 8c and Figure 9c. In contrast, the contents of Fe and Mo within these particles were relatively lower than those in the surrounding matrix, as shown in Figure 8e,f and Figure 9e,f. This indicates that the precipitates formed during tempering are primarily Cr-rich carbides, which play an important role in precipitation strengthening and contribute to the secondary hardening behavior observed near 550 °C.

### 3.4. TEM Analysis

**Figure 10:**
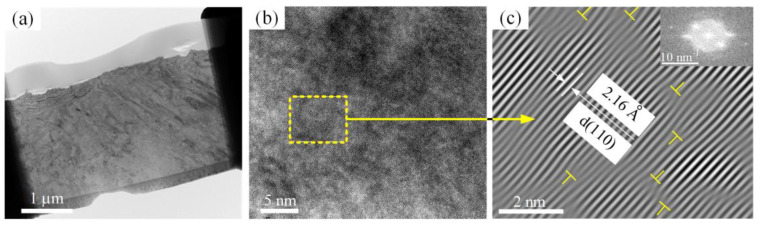
FIB-prepared TEM characterization of the as-quenched specimen: (**a**) microstructure of the TEM specimen prepared by FIB; (**b**) HRTEM image; (**c**) IFFT analysis result.

TEM specimens were prepared from the as-quenched sample using the FIB function of the SEM, followed by detailed TEM characterization. Distinct lath martensitic features were clearly observed, with lath widths ranging from approximately 200 to 500 nm, as shown in Figure 10a.

Further high-resolution TEM (HRTEM) analysis revealed clear lattice fringes. After inverse fast Fourier transform (IFFT) processing of the selected region in Figure 10b, multiple dislocations were identified. The measured lattice spacing was 0.216 nm, corresponding to the (110) crystal plane. The FFT diffraction spots appeared slightly blurred, indicating the presence of residual stress, as shown in Figure 10c. Clear and symmetrical diffraction spots suggest that the selected region possessed a well-ordered crystal lattice structure. Meanwhile, the diffuse background and broadened primary diffraction spots indicate the presence of crystal defects in localized regions. In addition, the elongation of diffraction spots suggests the existence of lattice distortion, as shown in the upper right corner of Figure 10c. These results indicate that the as-quenched martensitic structure contains a high density of crystal defects and internal stress, which provides an important structural basis for the subsequent tempering response and mechanical property evolution.

**Figure 11:**
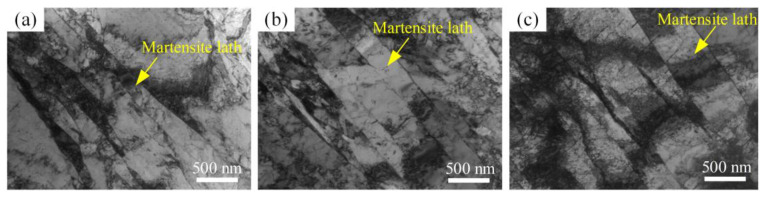
Analysis of lath microstructures: (**a**) tempered at 200 °C; (**b**) tempered at 400 °C; (**c**) tempered at 600 °C.

The microstructural evolution of martensitic structure at different tempering temperatures was further analyzed by TEM. After tempering at 200 °C, a large amount of clear lath martensite was retained in the microstructure, as shown in Figure 11a. At 400 °C, the martensitic laths began to coarsen, as shown in Figure 11b. After tempering at 600 °C, the lath structure became significantly coarser, as shown in Figure 11c. Overall, from 200 °C to 400 °C and then to 600 °C, the width of laths increased progressively.

**Figure 12:**
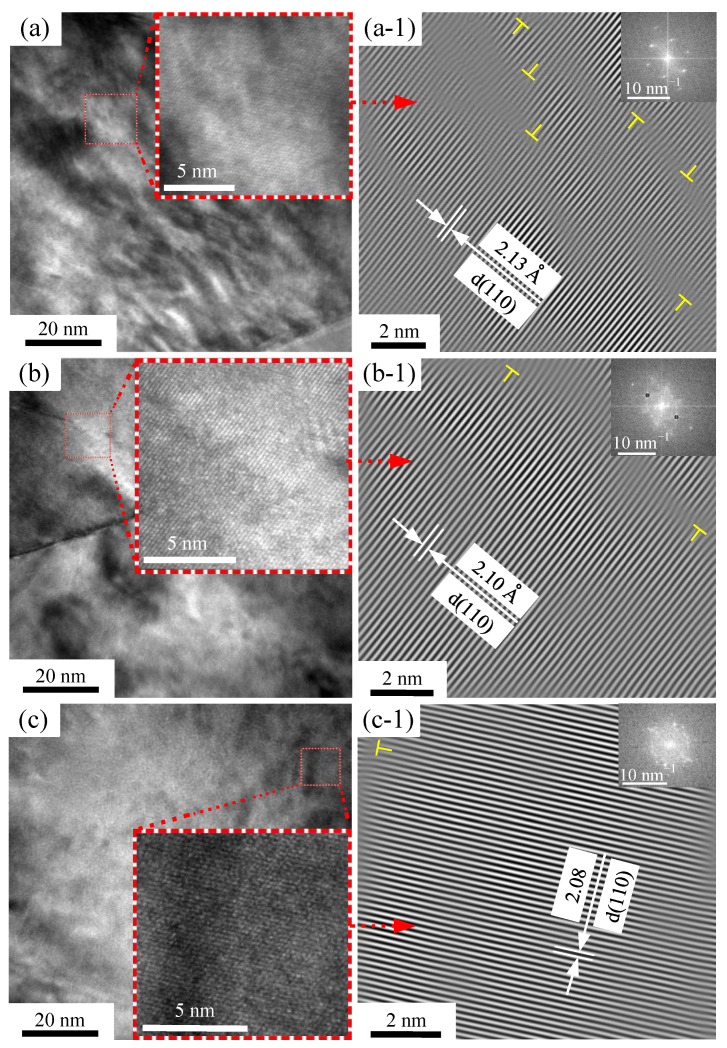
HRTEM analysis of specimens tempered at different temperatures: (**a**–**c**) HRTEM images of specimens tempered at 200 °C, 400 °C, and 600 °C, respectively; (**a-1**–**c-1**) corresponding IFFT results of the selected local regions in (**a**–**c**), respectively.

Figure 12a, Figure 12b and Figure 12c show the HRTEM images of the specimens tempered at 200 °C, 400 °C, and 600 °C, respectively. Figure 12(a-1), Figure 12(b-1), and Figure 12(c-1) present the corresponding IFFT images extracted from the red dashed regions in Figure 12a, Figure 12b and Figure 12c, respectively. In Figure 12(a-1), the measured lattice fringe spacing is 0.213 nm, corresponding to the (110) crystallographic plane [17]. Similarly, the lattice spacings in Figure 12(b-1) and Figure 12(c-1) are 0.210 nm and 0.208 nm, respectively, both corresponding to the (110) plane. Regarding dislocation characteristics, the specimen tempered at 200 °C exhibits more pronounced dislocation contrast and a denser dislocation configuration compared with those tempered at higher temperatures. With increasing tempering temperature, the dislocation structures become progressively relaxed, indicating that dislocation rearrangement and recovery occur during tempering. Meanwhile, the gradual reduction in lattice fringe spacing suggests a decrease in lattice distortion associated with carbon redistribution and carbide precipitation during tempering.

**Figure 13:**
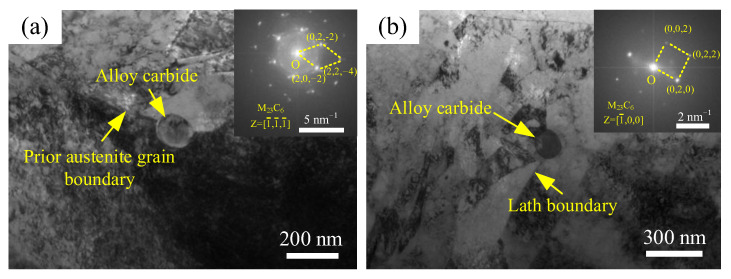
TEM microstructures of the specimen tempered at 550 °C: (**a**) precipitation of alloy carbides along prior austenite grain boundaries; (**b**) precipitation of alloy carbides along martensitic lath boundaries.

TEM observations of the specimen tempered at 550 °C reveal the presence of fine precipitates along prior austenite grain boundaries, as shown in Figure 13a. The corresponding diffraction pattern, displayed in the upper-right inset of Figure 13a, confirms that these precipitates possess an M23C6-type structure. Similar fine precipitates are also observed at martensitic lath boundaries, as shown in Figure 13b. Combined with the previous EDS analysis, these particles are identified as Cr-rich alloy carbides. The precipitation of these carbides along grain boundaries and lath boundaries plays a crucial role in impeding dislocation motion and inhibiting boundary migration, thereby making a significant contribution to the secondary hardening observed at 550 °C.

### 3.5. Fracture Morphology Analysis of Impact Specimens

**Figure 14:**
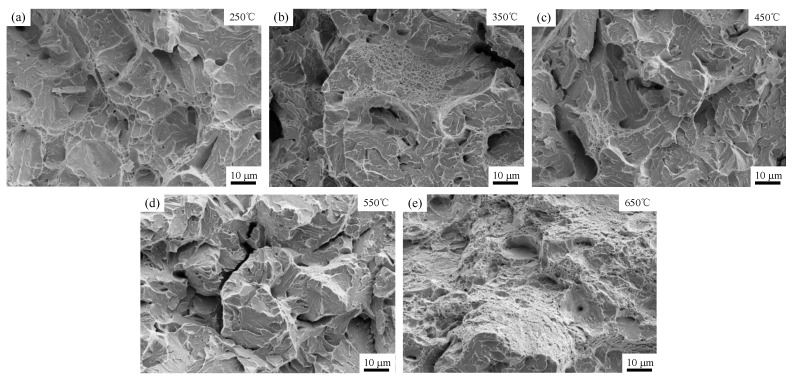
SEM images of Charpy impact fracture surfaces of specimens tempered at different temperatures: (**a**) 250 °C; (**b**) 350 °C; (**c**) 450 °C; (**d**) 550 °C; (**e**) 650 °C.

To further understand the variation in impact toughness with tempering temperature, the fracture surfaces of Charpy impact specimens were examined by scanning electron microscopy (SEM). The fracture morphologies of specimens tempered at different temperatures are shown in Figure 14. The fracture characteristics exhibit a clear evolution with increasing tempering temperature, which is consistent with the variation trend of impact toughness shown in Figure 4.

At 250 °C, the fracture surface mainly consists of relatively flat facets, cleavage-like regions, and tearing ridges, indicating a limited plastic deformation capability during impact fracture. This behavior is associated with the high dislocation density and residual stresses retained in the quenched martensitic structure, which increase the resistance to plastic deformation and promote crack propagation.

With increasing tempering temperature to 350 °C and 450 °C, the fracture surfaces become progressively rougher, accompanied by the appearance of more ductile features such as fine dimples. The increased proportion of dimpled regions suggests that tempering-induced stress relaxation and dislocation recovery improve the plastic accommodation capability of the martensitic matrix, resulting in enhanced impact toughness.

At 550 °C, although the material exhibits the highest hardness and tensile strength due to secondary hardening, the fracture surface still contains a considerable fraction of quasi-cleavage features. This indicates that the formation of finely dispersed alloy carbides improves strength through effective dislocation pinning, but simultaneously restricts dislocation mobility and plastic deformation during impact loading. Therefore, the strengthening effect is accompanied by a reduction in impact toughness.

When the tempering temperature increases to 650 °C, the fracture surface exhibits abundant dimples and tearing features, indicating an enhanced ductile fracture tendency. The improved plastic deformation capability is attributed to intensified dislocation recovery and carbide coarsening, which reduce the obstruction to dislocation motion and facilitate plastic deformation before fracture. These fracture characteristics provide direct evidence for the strength–toughness trade-off behavior during tempering.

## 4. Discussion

### 4.1. Mechanism of Hardness and Tensile Strength Evolution

Overall, as the tempering temperature increased from 200 °C to 650 °C, the hardness and tensile strength of 1Cr12Ni2WMoVNb steel first decreased slightly, then increased to a secondary hardening peak, and finally declined rapidly. The secondary hardening peak occurred at 550 °C, as shown in Figure 4.

**Figure 15:**
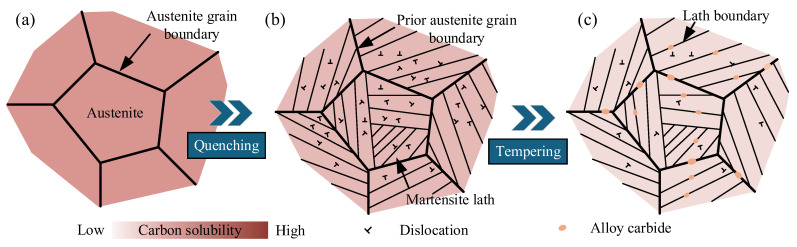
Schematic illustration of the microstructural evolution of the steel during quenching and tempering: (**a**) austenitic structure at high temperature; (**b**) martensitic structure after quenching; (**c**) tempered microstructure after carbide precipitation.

During quenching, after austenitization followed by rapid cooling, the crystal structure transforms from γ-Fe to α-Fe through a diffusionless shear transformation, accompanied by the transformation of austenite into martensite. This process produces lath-shaped body-centered cubic martensite containing supersaturated solid-solution carbon, resulting in severe lattice distortion and a significant increase in dislocation density, thereby markedly improving the hardness and strength of the material. Various crystal defects in the microstructure, such as dislocations, grain boundaries, and carbide precipitates, have a significant influence on the mechanical properties. Appropriate defect structures can effectively enhance strength through dislocation pinning, solid-solution strengthening, grain refinement, and precipitation strengthening mechanisms. However, excessive or non-uniform defects may generate high internal stresses, which can act as crack initiation sites and reduce the overall mechanical performance. Therefore, tempering is an essential process for relieving quenching-induced internal stress and optimizing the balance between strength and toughness. During tempering, alloy carbides preferentially precipitate along prior austenite grain boundaries and martensitic lath boundaries, as shown in Figure 15.

During the tempering process, alloying elements such as Cr, Mo, and V combine with carbon to form finely dispersed alloy carbides, including M23C6-, M7C3- and MC-type carbides. The initial precipitation of these carbides contributes significantly to strengthening by effectively hindering dislocation motion. However, with a further increase in tempering temperature, these carbides gradually coarsen, leading to larger particle sizes and a reduced number density [18]. As a result, the interfacial bonding strength between the coarsened carbides and the matrix weakens, significantly reducing the precipitation strengthening effect. In addition, carbide coarsening decreases the solute concentration in the matrix, further weakening the solid-solution strengthening effect [19].

Meanwhile, the martensitic structure gradually decomposes, and the original lath martensite progressively transforms into coarsened tempered sorbite, accompanied by a significant reduction in dislocation density [20]. This microstructural evolution facilitates dislocation motion and weakens the resistance to plastic deformation, ultimately leading to a decrease in hardness and tensile strength. The formation of tempered sorbite shown in Figure 6i,j provides direct evidence for this process. Therefore, the combined effects of solid-solution strengthening, dislocation strengthening, grain refinement strengthening, and precipitation strengthening jointly govern the evolution of hardness and tensile strength during tempering.

#### 4.1.1. Analysis of Solid-Solution Strengthening

Solid-solution strengthening occurs when solute atoms enter the matrix lattice, causing lattice distortion and generating elastic stress fields that interact with dislocations, thereby increasing the resistance to dislocation motion. For martensitic stainless steel, interstitial solid-solution strengthening is mainly contributed by C and N atoms, while substitutional atoms such as Cr, Mo, and V provide substitutional solid-solution strengthening. The strengthening effect can be described by the following equation [21,22]:

(1) $$∆\sigma_{ss}=k_{i}C_{i}^{n}$$

where $∆\sigma_{ss}$ represents the increment of solid-solution strengthening, $k_{i}$ is the strengthening coefficient, $C_{i}^{n}$ is the solid-solution concentration of a specific element, i deno tes a specific alloying element, and n is the strengthening exponent. For interstitial solute atoms such as C and N, n generally ranges from 0.33 to 2.0, whereas for substitutional solute atoms such as Mo, Si, and Mn, n is usually between 0.5 and 1.0 [23,24]. According to Equation (1), the solid-solution strengthening contribution is strongly dependent on the concentration of solute atoms remaining in the matrix. During tempering, the precipitation of carbides reduces the concentration of dissolved carbon atoms, resulting in a decrease in $∆\sigma_{ss}$ and contributing to the reduction in hardness and tensile strength shown in Figure 4.

When solute atoms dissolve into the ferritic matrix, lattice distortion occurs and elastic stress fields are generated. The interaction between these stress fields and moving dislocations hinders dislocation motion, thereby producing a strengthening effect. Among all solute atoms, the interstitial strengthening effect of C and N is the most significant. When multiple alloying elements are simultaneously dissolved in solid solution, their strengthening effects usually exhibit a synergistic superposition. In addition, solute atoms not only increase the critical stress required for dislocation slip, but may also influence the precipitation behavior of carbides or nitrides, thereby indirectly affecting subsequent precipitation strengthening.

**Figure 16:**
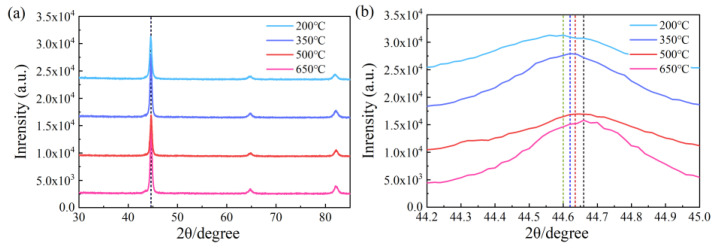
XRD analysis of 1Cr12Ni2WMoVNb steel tempered at different temperatures: (**a**) XRD patterns of specimens tempered at different temperatures; (**b**) enlarged view of the (110) diffraction peak region shown in (**a**).

Phase analysis was performed on specimens tempered at 200 °C, 350 °C, 500 °C, and 650 °C, as shown in Figure 16a. The results show that the (110) diffraction peak of the specimen tempered at 200 °C was located at 44.60°. With increasing tempering temperature, the (110) peak gradually shifted toward higher diffraction angles, as shown in Figure 16b. In X-ray diffraction analysis, the diffraction peak position is determined by Bragg’s law [25]:

(2) $$2d_{hkl}sin\theta =n\lambda$$

where $d_{hkl}$ is the interplanar spacing, θ is the diffraction angle, λ is the X-ray wavelength, and n is the diffraction order (generally taken as n = 1). For body-centered cubic (BCC) martensite, the interplanar spacing can be calculated by [26]

(3) $$d_{hkl}=\frac{a}{\sqrt{h^{2}+k^{2}+l^{2}}}$$

where a is the lattice constant, and h, k, and l are the Miller indices of the crystal plane. It can be seen that changes in the lattice constant a directly affect the interplanar spacing $d_{hkl}$, thereby causing variations in the diffraction angle θ and the diffraction peak position. When carbon atoms are dissolved interstitially in the martensitic lattice, they induce lattice distortion and increase the lattice constant, resulting in an increase in $d_{hkl}$. According to Bragg’s law, this leads to a decrease in the diffraction angle θ, which is manifested as a shift in the diffraction peak toward lower angles [27].

**Figure 17:**
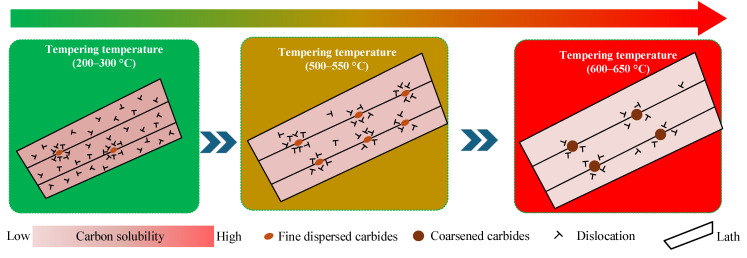
Schematic illustration of the microstructural evolution with increasing tempering temperature.

Based on the phase analysis results, the (110) diffraction peak gradually shifts toward higher angles with increasing tempering temperature. This shift suggests a reduction in lattice distortion during tempering, which is associated with carbon redistribution from the supersaturated martensitic matrix and relaxation of lattice strain [28]. During tempering, carbides preferentially nucleate and precipitate at crystal defects, such as prior austenite grain boundaries and martensitic lath boundaries. According to Equation (1), the reduction of carbon concentration in the martensitic matrix directly decreases the solid-solution strengthening contribution ($∆\sigma_{ss}$). Therefore, the gradual decrease in lattice distortion revealed by XRD results provides microstructural evidence for the reduction in solid-solution strengthening, which contributes to the decrease in hardness and tensile strength during the early tempering stage, as shown in Figure 17. Furthermore, as shown in Figure 12(a-1–c-1), the interplanar spacing of the (110) planes decreases progressively with increasing tempering temperature, which further corroborates the reduction in lattice distortion and the diminished carbon solubility.

Meanwhile, the precipitated carbide particles can enhance strength by exerting a pinning effect on dislocations, thereby restricting dislocation motion. This precipitation strengthening effect can partially compensate for the strength loss caused by the reduction in solid-solution strengthening, enabling dynamic regulation of material strength during tempering. Furthermore, due to the regulating effects of alloying elements such as Cr and Mo on the precipitation kinetics and stability of carbides in martensitic stainless steel, a typical secondary hardening peak appears with increasing tempering temperature.

#### 4.1.2. Analysis of Dislocation Strengthening

Dislocation strengthening is one of the most common strengthening mechanisms during the plastic deformation of metals. As strain increases, the dislocation density continuously rises, and dislocations interact, entangle, and intersect to form complex dislocation networks, which effectively hinder the subsequent motion of dislocations. This strengthening effect is usually described by the following equations [27]:

(4) $$∆\sigma_{D}=k_{D}\rho^{1/2}$$

(5) $$k_{D}=2\alpha Gb$$

where $∆\sigma_{D}$ represents the increment of dislocation strengthening, $k_{D}$ is the strengthening coefficient, and ρ is the dislocation density. In this study, α = 0.20, b = 0.248 nm, and G represents the shear modulus [28]. According to Equations (4) and (5), the dislocation strengthening contribution ($∆\sigma_{D}$) is proportional to the square root of the ρ. Therefore, the recovery process during tempering, including dislocation annihilation and rearrangement, reduces ρ and consequently decreases $∆\sigma_{D}$. This reduction in dislocation strengthening contributes to the gradual decrease in hardness and tensile strength observed in Figure 4, particularly during the early tempering stage. The TEM images in Figure 12(a-1–c-1) show a gradual decrease in dislocation density with increasing tempering temperature, which is consistent with the above analysis.

For martensitic steels with a BCC structure, dislocation motion is inherently restricted by a relatively high Peierls stress [29]. This crystallographic characteristic makes dislocation slip more difficult, causing dislocation strengthening to play a more dominant role in the overall strengthening mechanism of the material. However, dislocation strengthening also involves a significant trade-off in mechanical properties. When the dislocation density becomes excessively high, severe dislocation entanglement and mutual obstruction significantly reduce the plastic deformation capability of the material. Meanwhile, the ductile-to-brittle transition temperature increases markedly, which adversely affects the low-temperature service reliability of martensitic steels. Therefore, controlling the evolution of dislocation density during tempering is essential for achieving an optimal balance between strength and toughness.

#### 4.1.3. Analysis of Grain Refinement Strengthening

Grain boundaries act as natural barriers to dislocation motion and play a particularly important role in fine-grained materials. The smaller the grain size, the larger the total grain boundary area, and the more pronounced the dislocation pile-up effect becomes. The classical quantitative description of grain refinement strengthening is given by the Hall–Petch relationship [30,31]:

(6) $$∆\sigma_{G}=k_{y}d^{-1/2}$$

where d is the average grain diameter, $∆\sigma_{G}$ represents the strengthening increment induced by grain refinement, and $k_{y}$ is a material-dependent constant.

SEM observations revealed well-defined prior austenite grain boundaries, with an average grain size ranging from ~42 to 110 μm, as indicated by the yellow arrows in Figure 6a–j. The martensitic lath morphology was clearly delineated after etching, as highlighted by the red arrows. Moreover, the lath size in Figure 7f–k is evidently larger than that in Figure 7a–e, accompanied by a noticeable reduction in the lath aspect ratio. These observations are in good agreement with the TEM results (Figure 11).

The coarsening of lath martensite with increasing tempering temperature is primarily governed by dislocation recovery and boundary migration. The lath boundaries, composed of high-density dislocation arrays, progressively lose their stability due to dislocation annihilation and rearrangement at elevated temperatures [32]. Meanwhile, enhanced atomic diffusion promotes dislocation climb and accelerates boundary migration, thereby reducing the interfacial energy [33,34]. As a result, smaller laths are gradually consumed by larger ones, leading to overall lath coarsening. In addition, the precipitation and subsequent coarsening of carbides weaken the pinning effect on lath boundaries, further facilitating boundary migration and accelerating the coarsening process.

These results indicate that, with increasing tempering temperature, the martensitic lath structure undergoes progressive coarsening, resulting in an increase in the effective grain size (d). According to the Hall–Petch relationship (Equation (6)), the grain refinement strengthening contribution ($∆\sigma_{G}$) decreases with increasing d. Therefore, the reduction in effective boundary strengthening caused by lath coarsening contributes to the decrease in hardness and tensile strength observed in Figure 4.

#### 4.1.4. Analysis of Precipitation Strengthening

Second-phase particles strengthen the material by pinning dislocations through either cutting or bypassing mechanisms. During the tempering of martensitic steels, finely dispersed carbides commonly precipitate and contribute to strengthening by hindering dislocation motion. The precipitation strengthening mechanism can be qualitatively described by the following relationship [35]:

(7) $$∆\sigma_{P}=k_{P}\lambda^{-1/2}$$

where $∆\sigma_{P}$ represents the increment of precipitation strengthening, $k_{P}$ is a material constant, and λ is the interparticle spacing of the second-phase particles. According to Equation (7), a reduction in the interparticle spacing (λ) of precipitates increases the precipitation strengthening contribution ($∆\sigma_{P}$). Therefore, the formation of fine and uniformly distributed carbides during tempering can effectively compensate for the weakening of solid-solution and dislocation strengthening.

In addition to directly impeding dislocation motion, precipitated phases can also stabilize the martensitic matrix and suppress recovery and recrystallization during thermal exposure, thereby enhancing the mechanical stability of the material at elevated temperatures. These precipitates mainly originate from phase transformations occurring during the quenching and tempering processes.

Combined with the microstructural observations in Figure 6, it is evident that, compared with other tempering temperatures, the number of fine particles significantly increases in the specimens tempered at 500 °C and 550 °C, as indicated by the red arrows in Figure 6g,h. Further EDS analyses in Figure 8 and Figure 9 reveal that these particles are enriched in Cr, while the TEM-based structural characterization in Figure 13 suggests that these particles correspond to M23C6-type carbides. Therefore, these fine particles are identified as alloy carbides precipitated during the tempering process. In 1Cr12Ni2WMoVNb steel, the presence of Cr, Mo, V, and Nb provides a complex carbide precipitation system. During tempering, the redistribution of these alloying elements promotes the formation of stable carbide particles, which produces a stronger precipitation-strengthening response compared with conventional low-alloy martensitic steels. Therefore, the strength evolution is not solely determined by carbide precipitation, but by the competition between carbide strengthening and the simultaneous loss of solid-solution and dislocation strengthening.

In particular, at 550 °C, the precipitation of fine and uniformly distributed alloy carbides becomes most pronounced. According to Equation (7), the reduced interparticle spacing and effective dislocation pinning provided by these carbides significantly increase the precipitation strengthening contribution ($∆\sigma_{P}$). Consequently, the enhancement of precipitation strengthening exceeds the softening effects caused by reduced solid-solution and dislocation strengthening, resulting in the secondary hardening behavior observed in Figure 4. These carbides effectively pin dislocations and inhibit plastic deformation, resulting in a marked increase in hardness and tensile strength, as shown in Figure 17.

Therefore, the yield strength ($\sigma_{y}$) of martensitic stainless steel is governed by the combined contributions of multiple strengthening mechanisms, including solid-solution strengthening, dislocation strengthening, grain boundary strengthening, and precipitation strengthening. The overall strengthening effect can be quantitatively described using either a linear superposition model or a root mean square (RMS) model [36,37]:

(8) $$\sigma_{y}=∆\sigma_{0}+∆\sigma_{G}+∆\sigma_{SS}+∆\sigma_{D}+∆\sigma_{P}$$

(9) $$\sigma_{y}=\sqrt{{\left(∆\sigma_{D}\right)}^{2}+{\left(∆\sigma_{G}+∆\sigma_{SS}+∆\sigma_{0}+∆\sigma_{P}\right)}^{2}}$$

These models indicate that the final strength evolution during tempering is determined by the competition among different strengthening mechanisms. At low tempering temperatures, the decrease in solid-solution and dislocation strengthening dominates, resulting in strength reduction. With increasing tempering temperature, the enhanced precipitation strengthening contribution gradually compensates for these weakening effects, leading to the strength recovery and secondary hardening peak near 550 °C.

#### 4.1.5. Mechanism of Secondary Hardening

1Cr12Ni2WMoVNb is a typical secondary hardening steel, and a pronounced secondary hardening phenomenon occurs during tempering. This behavior is mainly attributed to the specific evolution of microstructure and chemical composition during the tempering process. During tempering, solute atoms such as Cr, Mo, and V are gradually redistributed from the martensitic matrix and form alloy-enriched regions [38]. When the tempering temperature reaches approximately 550 °C, Cr-rich alloying elements precipitate and combine with carbon to form finely dispersed alloy carbides, mainly identified as M23C6-type carbides in this study. The precipitation of Cr-rich particles is further confirmed by the results shown in Figure 8, Figure 9 and Figure 13.

According to Equation (7) [39], the precipitation strengthening contribution ($∆\sigma_{P}$) is inversely related to the interparticle spacing (λ) of the precipitates. At the secondary hardening peak temperature, the finely dispersed alloy carbides exhibit a small interparticle spacing, resulting in an enhanced precipitation strengthening contribution. Consequently, the precipitation strengthening increment $∆\sigma_{p}$ becomes significantly larger. According to Equation (8) [40] and the following relationship [41]:

(10) $$Hv\approx 3\cdot \sigma_{y}$$

an increase in yield strength $\sigma_{y}$ directly leads to an increase in hardness Hv. These finely dispersed alloy carbides effectively pin dislocations and inhibit dislocation motion [17,42]. Therefore, the increase in precipitation strengthening compensates for the reduction in solid-solution and dislocation strengthening during tempering, leading to an increase in yield strength. According to Equation (10), the enhanced yield strength directly contributes to the increase in hardness, resulting in the secondary hardening peak near 550 °C observed in Figure 4. Meanwhile, excessive carbide coarsening at higher tempering temperatures reduces the precipitation strengthening effect and contributes to the subsequent decrease in strength.

It should be noted that the secondary hardening peak observed at approximately 550 °C corresponds to the maximum strength condition within the investigated temperature range. The exact peak temperature is closely related to carbide precipitation kinetics and alloying element redistribution, which requires further investigation using a narrower tempering interval.

### 4.2. Mechanism of Impact Toughness Evolution

During the tempering of alloy steels, the relationship between impact toughness and strength exhibits a complex evolution behavior, which is fundamentally governed by the competition among carbide precipitation strengthening, dislocation recovery, residual stress relief, and temper embrittlement. After quenching, the microstructure is predominantly composed of lath martensite characterized by a high dislocation density, supersaturated carbon, and significant residual stresses [43]. Under these conditions, the material exhibits high hardness and strength but limited plastic deformation capability, resulting in low impact toughness.

With increasing tempering temperature, in the low-temperature tempering regime (approximately 200–400 °C), the increase in impact toughness accompanied by the decrease in hardness and tensile strength (Figure 4) indicates the occurrence of tempering-induced softening. This behavior suggests the relaxation of quenching-induced residual stresses and the recovery of the martensitic matrix, which improve the plastic deformation capability of the material [44,45]. Furthermore, TEM observations in Figure 12(a-1–c-1) reveal a gradual reduction in dislocation density with increasing tempering temperature, providing direct microstructural evidence for the occurrence of recovery during tempering. According to the established tempering behavior of martensitic steels, the decomposition of supersaturated martensite and the formation of transition carbides may also occur within this temperature range [46]. These processes reduce lattice distortion and internal stress, while the accompanying loss of dislocation strengthening contributes to the slight decrease in strength but promotes toughness enhancement.

When the tempering temperature further increases to approximately 500–600 °C, alloying elements such as Cr precipitate to form finely dispersed alloy carbides (e.g., M23C6) [47]. These fine and stable precipitates strongly impede dislocation motion, giving rise to pronounced precipitation strengthening and a corresponding increase in strength and hardness (i.e., secondary hardening) [48]. For 1Cr12Ni2WMoVNb steel, the same carbide precipitation process responsible for secondary hardening also introduces a limitation on plastic accommodation. The dense distribution of alloy carbides increases resistance to dislocation motion, which improves strength but reduces the capacity for plastic deformation during impact loading [49]. This intrinsic competition explains the observed strength–toughness trade-off.

When the tempering temperature is further increased above ~600 °C, the dominant microstructural processes shift from precipitation strengthening to recovery, coarsening, and boundary evolution. The dislocation density is significantly reduced due to enhanced recovery, and the martensitic lath structure progressively breaks down, leading to a more equiaxed microstructure [50]. Meanwhile, the previously formed carbides undergo coarsening and their interparticle spacing increases, which weakens the precipitation strengthening effect [51,52]. Consequently, the resistance to dislocation motion decreases, allowing for enhanced plastic deformation. As a result, impact toughness increases markedly, despite the concurrent reduction in strength and hardness.

Therefore, the evolution of strength and impact toughness during tempering is governed by the dynamic competition between strengthening mechanisms and plastic deformation capability. At intermediate temperatures, precipitation strengthening and dislocation pinning enhance strength but restrict plastic deformation, thereby reducing impact toughness. In contrast, at higher tempering temperatures, the reduction in dislocation density and the coarsening of carbides restore plasticity, leading to a significant improvement in impact toughness despite the loss of strength. Under unfavorable conditions, grain boundary degradation may further dominate, resulting in temper embrittlement and a deterioration in toughness [53].

Thus, the inverse relationship between strength and toughness arises from the intrinsic trade-off between resistance to dislocation motion and the ability to accommodate plastic deformation during crack initiation and propagation.

## 5. Conclusions

The present study demonstrates the influence of tempering temperature on the microstructure evolution and mechanical properties of 1Cr12Ni2WMoVNb martensitic stainless steel. The variation in strength and toughness during tempering is associated with the competition between strengthening mechanisms and microstructural softening processes. The main conclusions are summarized as follows:

1. With increasing tempering temperature from 200 to 650 °C, the hardness and tensile strength initially decrease, followed by an increase to the maximum value near 550 °C, and subsequently decrease at higher temperatures. In contrast, the impact toughness exhibits an opposite evolution trend, indicating a clear strength–toughness trade-off during tempering.
2. At 300 °C tempering, the material achieves a relatively balanced combination of strength and toughness, with a hardness of 440.74 HV0.1, tensile strength of 1224.39 MPa, and impact toughness of 79.22 J. This condition provides a potential reference for applications requiring a balance between mechanical strength and deformation resistance.
3. The evolution of mechanical properties is closely related to the competition among different microstructural mechanisms. At lower tempering temperatures, recovery-induced reduction in dislocation strengthening and carbon redistribution-induced weakening of solid-solution strengthening contribute to strength degradation. At intermediate temperatures, enhanced carbide precipitation compensates for these softening effects, resulting in strength recovery.
4. The secondary hardening behavior observed near 550 °C is associated with the formation of fine and dispersed alloy carbides. TEM and compositional analyses suggest that these precipitates are mainly Cr-rich carbides with an M23C6-type structure. Their interaction with dislocations enhances resistance to plastic deformation and contributes to the increase in hardness and tensile strength.
5. At higher tempering temperatures above approximately 600 °C, accelerated dislocation recovery and carbide coarsening weaken the strengthening contribution, while the improved plastic deformation capability leads to a significant increase in impact toughness accompanied by reduced strength.

## Figures and Tables

**Table 1:** Chemical composition of 1Cr12Ni2WMoVNb steel [16] (wt.%).

Content	C	Cr	Ni	Mo	V	W	Si	Mn	Nb	P	S
Minimum	0.11	11	1.8	0.8	0.2	0.7	–	–	0.15	–	–
Maximum	0.17	12	2.2	1.2	0.3	1	0.6	0.6	0.3	0.03	0.025

## Data Availability

The original contributions presented in this study are included in the article. Further inquiries can be directed to the corresponding author.
